# PD-1/PD-L1 Inhibitors in Patients With Preexisting Autoimmune Diseases

**DOI:** 10.3389/fphar.2022.854967

**Published:** 2022-03-18

**Authors:** Ke Zhang, Xiangyi Kong, Yuan Li, Zhongzhao Wang, Lin Zhang, Lixue Xuan

**Affiliations:** ^1^ Department of Breast Surgical Oncology, National Cancer Center/National Clinical Research Center for Cancer/Cancer Hospital, Chinese Academy of Medical Sciences and Peking Union Medical College, Beijing, China; ^2^ School of Population Medicine and Public Health, Chinese Academy of Medical Sciences and Peking Union Medical College, Beijing, China; ^3^ Melbourne School of Population and Global Health, the University of Melbourne, Melbourne, VIC, Australia; ^4^ Centre of Cancer Research, Victorian Comprehensive Cancer Centre, Melbourne, VIC, Australia

**Keywords:** PD-1/PD-L1 inhibitors, autoimmune diseases, cancer, immune-related adverse events, immunotherapy

## Abstract

Autoimmune diseases and malignant tumors are the two hotspots and difficulties that are currently being studied and concerned by the medical field. The use of PD-1/PD-L1 inhibitors improves the prognosis of advanced tumors, but excessive immune responses can also induce immune-related adverse events (irAEs). Due to this concern, many clinical trials exclude cancer patients with preexisting autoimmune disease (AID). This review outlines the possible mechanisms of irAE, discusses the safety and efficacy of PD-1/PD-L1 inhibitors in cancer patients with preexisting AID, and emphasizes the importance of early recognition, continuous monitoring, and multidisciplinary cooperation in the prevention and management of cancer patients with preexisting AID.

## Introduction

**FIGURE 1:**
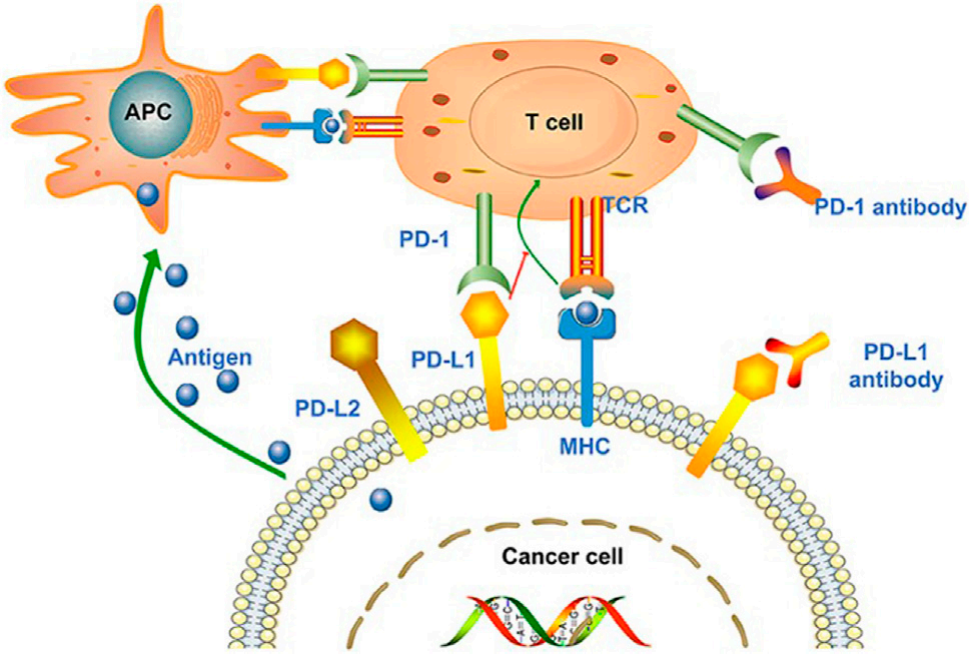
Tumor cell-mediated immune escape and the treatability of PD-1/PD-L1 inhibitors. Antigen-presenting cells (APCs) recognize the antigens released by tumor cells and present them to T cells to promote the activation of T cell. However, the ligand PD-L1 expressed on tumor cells binds to PD-1 on T cells, promoting T cell dysfunction and inhibiting immune response. In the context of MHC, tumor cell antigens can also be presented directly to activate T cells. PD-1/PD-L1 inhibitors can block the binding of T cells to tumor cells and inhibit immune evasion. Reprinted from Su et al. (2020). Copyright ^©^ 2020 Su, Wang, Liu, Guo, Zhang, Li, Zhou, Yan, Zhou, and Zhang.

Programmed cell death protein 1 receptor (PD-1), also known as CD279, is a type I transmembrane protein receptor containing 288 amino acids. It was first described in the early 1990s and is expressed during apoptosis induction in T cell hybridomas (Gong et al., 2018). Subsequent reports found that PD-1-deficient mice exhibited autoimmune disease-like features (lupus-like arthritis, glomerulonephritis, and splenomegaly), demonstrating that PD-1 is a negative regulator of immune response (Nishimura et al., 1999). PD-1 is expressed on activated T cells, B cells, macrophages, regulatory T cells (Tregs), and natural killer (NK) cells. Binding to programmed death ligand 1 (PD-L1 or B7-H1) or PD-L2 (B7-DC) negatively regulates T cell-mediated immune responses in peripheral tissues to limit the effector T cell responses and protect the tissues from immune-mediated tissue damage (Keir et al., 2008). The interaction of PD-1/PD-L1 in the tumor microenvironment can promote T cell dysfunction, failure, apoptosis, neutralization, and the formation of IL-10, thus enhancing the proliferation and survival of tumor cells to promote the development and progression of cancer (Akinleye and Rasool, 2019). Studies have shown that tumors are highly infiltrated by Treg cells, and the co-inhibitory receptors expressed on Tregs (such as PD1) and a series of co-inhibitory ligands (such as PD-L1/PD-L2) can significantly promote tumor escape (Nishimura et al., 1999). In this context, PD-1/PD-L1 signal transduction represents a viable target for novel anticancer therapy. PD-1/PD-L1 inhibitors came into being and gradually became the focus of attention Figure 1.

Although PD-1/PD-L1 inhibitors have been shown to be more effective and less toxic than chemotherapy, immune-related adverse events (irAEs) that may be related to their mechanism have been observed (Okwundu et al., 2021). Common sites of irAE include the thyroid, gastrointestinal tract, skin, and liver, but any organ system can be affected (Okwundu et al., 2021). Although most irAEs are usually reversible or easily controllable, they may be associated with irreversible organ damage or death in rare cases (Wang et al., 2018).

Autoimmune diseases are generally considered relatively uncommon [the overall prevalence is about 3–5% (Wang et al., 2015) in the general population], but their impact on mortality and morbidity is significant. Under the influence of certain factors, the body’s tissue components or the immune system itself has some abnormalities, causing the immune system to mistakenly treat its own components as foreign objects to attack, that is, the destruction of immune tolerance, which is the basis of autoimmune diseases. IrAEs caused by PD-1/PD-L1 inhibitors usually have similar phenotypes and physiological characteristics to autoimmune diseases. For patients with preexisting AID, the use of immunotherapy seems to exacerbate the underlying autoimmune diseases (Kennedy et al., 2019). Therefore, cancer patients with autoimmune diseases have traditionally been excluded from most clinical trials. At present, the efficacy and safety of PD-1/PD-L1 inhibitors in cancer patients with AID remain unknown. There are also concerns about whether the frequent baseline use of immunosuppressants such as corticosteroids at the beginning of PD-1/PD-L1 inhibitors in AID patients may reduce efficacy in these patients; whether the status and types of autoimmune diseases will alter the risk of adverse events; and whether patients with underlying autoimmune diseases may benefit more from immunotherapy. These questions are still unknown yet. In this review, we summarized the relevant retrospective studies and several similar reviews from basic to clinical to discuss the mechanism of irAE and the safety and effectiveness of PD-1/PD-L1 inhibitors in AID patients and finally gave an overall overview of the prevention and management of adverse events, introduced some novel therapeutic methods, and looked forward to the future.

### Relationship Between Autoimmune Diseases and Cancer

**FIGURE 2:**
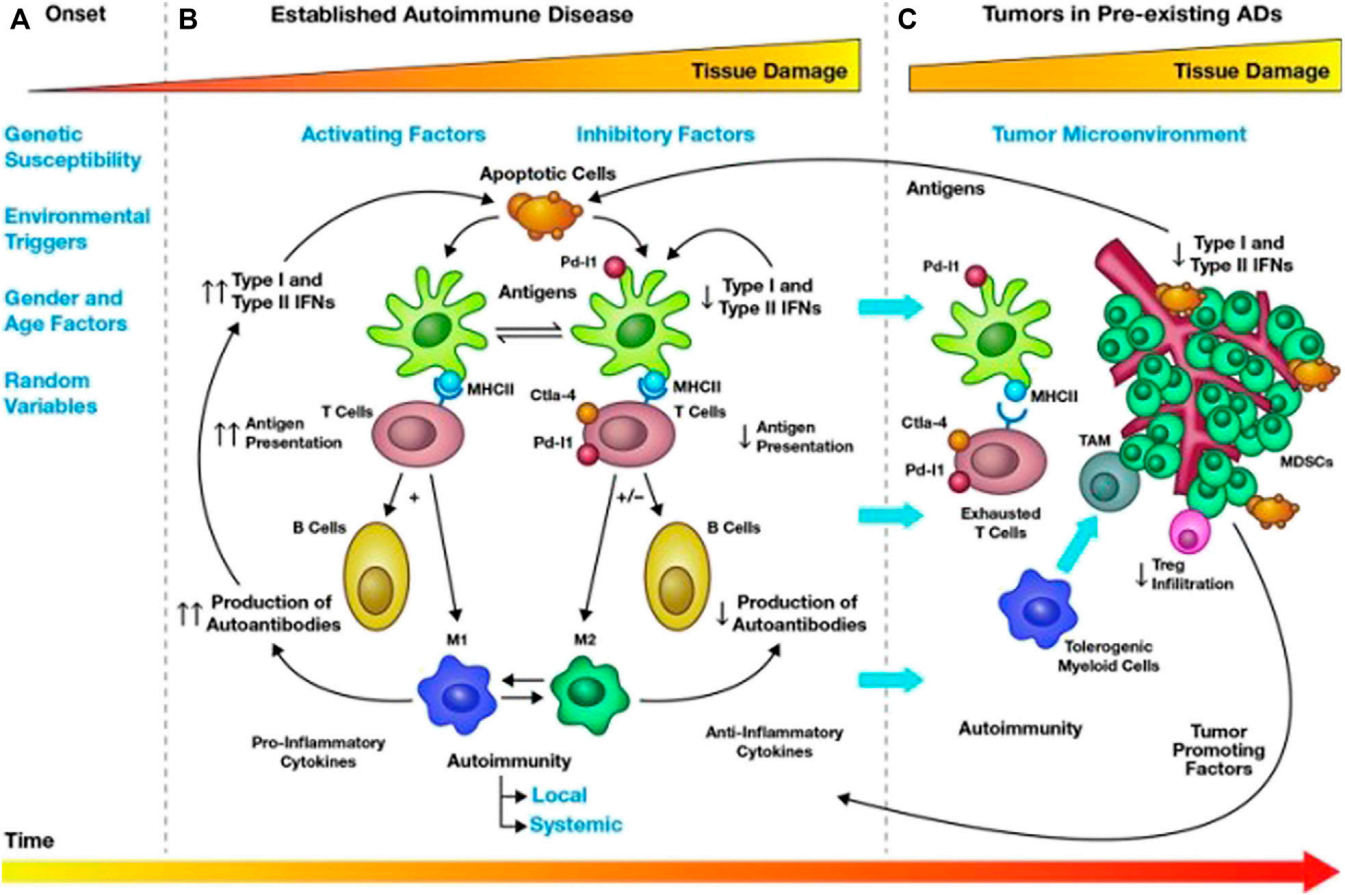
Mechanisms of cancer occurrence and regulation in autoimmune diseases. Tumorigenesis in AID can be divided into three stages **(A–C)**. In the first stage [**(A)** Onset], genetic predisposition, environment, gender, age, and random factors can contribute to the development of autoimmunity in a healthy individual, which lasts about three or 4 years. In the second stage [**(B)** Established autoimmune disease], as time goes on, a runaway mix of pro-inflammatory and anti-inflammatory events, maintaining the balance between co-activators (type I and II interferon) and co-suppressors (PD-1/PD-L1 and CTLA4) through responsive feedback pathways activated by T cells, B cells, dendritic cells, and macrophages, which is the characteristic of AID, can cause the damage of organs and tissues. At the same time, chronic inflammation may lead to a loss of antiproliferative signals and abnormal differentiation of normal cells, eventually leading to the development of cancer. In the third stage [**(C)** Tumor in preexisting AIDs], tumors regulate the internal environment of autoimmunity, evade immune surveillance through tumor-promoting factors, and break the balance of chronic inflammation by causing T cells depletion and immune tolerance through co-inhibitory molecules. Therefore, we need to use immunotherapy to curb tumor progression. CTLA-4, cytotoxic T lymphocyte-associated proteins 4; IFN, interferon; MDSCs, myeloid-derived suppressor cells; MHC, major histocompatibility complex; PD-L1, programmed cell death-1 ligand; TAM, tumor-associated macrophages. Reprinted from Valencia et al. (2019). Copyright ^©^ 2019, Mary Ann Liebert, Inc., publishers.

Cancer and autoimmune diseases are two different pathological conditions, especially in terms of immunity, showing opposite patterns, that is, cancer can evade the immune system or weaken the host’s immune response, while autoimmune diseases are the host’s immune response to self-antigens. However, there is plenty of evidence to suggest that the association between cancer and autoimmune diseases is bidirectional (Giat et al., 2017). On the one hand, the antitumor immune response may cross-react with the autologous tissue, leading to the development of autoimmunity (Masetti et al., 2021). On the other hand, Figure 2 suggests that an increased risk of malignancy was observed in different autoimmune diseases. This may be associated with chronic inflammation and tissue damage caused by autoimmunity, inability to clear carcinogenic viral infections, and long-term immunosuppressive therapy. In theory, chronic immunosuppression can promote the development and progression of malignant tumors, while enhanced immune function may reduce the incidence, progression, and aggressiveness of cancer. Therefore, clinicians may think that autoimmune diseases have a negative impact on tumor development, but this is precisely not true. The interaction between autoimmune and cancer is a complex multistep process.

Patients with AID have an increased risk of cancer, which is mainly related to hematological malignancies (Niccolai et al., 2020). Approximately 14%–25% (Khan et al., 2016; Haanen et al., 2020b) of patients diagnosed with lung cancer suffer from autoimmune diseases, including but not limited to rheumatoid arthritis (RA), psoriasis (PSO), polymyalgia rheumatica (PMR), systemic lupus erythematosus (SLE), inflammatory bowel disease (IBD), and Sjogren’s syndrome (SS). Next, we will review the cancer risk of some autoimmune diseases. RA: In the meta-analysis of Simon and colleagues (Simon et al., 2015), the standardized incidence rate (SIR) of lymphoma was 2.46 (95% CI, 2.05–2.96), malignant lymphoma was 3.21 (95% CI, 2.42–4.27), and non-Hodgkin lymphoma was 2.26 (95% CI, 1.82–2.81). The risk of lung cancer (SIR, 1.64; 95% CI, 1.51–1.79) and melanoma (SIR, 1.23; 95% CI, 1.01–1.49) has also increased. It is speculated that RA itself may cause a continuous inflammatory state, and cancer may have common risk factors with RA, which lead to increased cancer risk (Pundole and Suarez-Almazor, 2020). PSO: In a systematic review and meta-analysis of 112 studies including more than 2 million patients (Geller et al., 2018; Vaengebjerg et al., 2020), the overall prevalence of cancer in patients with psoriasis was 4.78% (95% CI, 4.02%–5.59%). The overall cancer risk increased slightly, with a risk ratio (RR) of 1.21 (95% CI, 1.11–1.33). The risk of several cancers is increased, including keratinocyte carcinoma (RR, 2.28; 95% CI, 1.73–3.01), lymphoma (RR, 1.56; 95% CI, 1.37–1.78), lung cancer (RR, 1.26; 95% CI, 1.13–1.40), and bladder cancer (RR, 1.12; 95% CI, 1.04–1.19). SLE: In 2015, Cao et al. (2015) included 16 studies involving 59,662 SLE patients and found that the overall RR of cancer was 1.28 (95% CI, 1.17–1.41). In 2018, Song et al. (2018) included 24 studies, and the results showed that SLE was associated with an increased overall cancer risk (SIR, 1.28; 95% CI, 1.16–1.42). The underlying mechanism may explain the development of cancer in SLE patients. On the one hand, patients have a basic deficiency in their immune function, leading to immune disorders, which may prevent abnormal cells from being removed and ultimately lead to an increased risk of cancer. On the other hand, drugs used for immunosuppressive therapy may also exacerbate immune disorders and further increase the risk of cancer. IBD: The risk of cancer in IBD patients is related to time. It increases by 2% in 10 years, 8% in 20 years, and 18% in 30 years (Eaden et al., 2001; Nadeem et al., 2020). In IBD patients, chronic intestinal inflammation is the main risk factor for gastrointestinal malignancies. Cancers caused by chronic intestinal inflammation include colorectal cancer (SIR, 5.7; 95% CI, 4.6–7.0), small intestinal adenocarcinoma (SIR, 27.1; 95% CI, 14.9–49.2), intestinal lymphoma (SIR, 17.51; 95% CI, 6.43–38.11), anal cancer, and cholangiocarcinoma (Axelrad et al., 2016). SS: In a systematic review (Cappelli and Shah, 2020), the overall cancer risk of Sjogren’s syndrome is higher than that of the general population, with a risk ratio of 1.53. The most common cancer is lymphoma. Patients with primary Sjogren’s syndrome (pSS) are 10–44 times more likely to develop lymphoma than healthy individuals (Igoe et al., 2020). In order to lay the foundation of risk stratification and targeted cancer screening, larger longitudinal cohort studies that provide a more detailed framework of the links between cancer and autoimmunity are urgently needed. In view of the increased risk of cancer in patients with autoimmune diseases, for clinicians, it is important to be aware of the cancer risk of a patient with autoimmune disease. At the same time, when receiving a cancer patient, it is necessary to distinguish whether the patient has previously had autoimmune diseases before making the next correct decision Table 1.

**TABLE 1:** Patients with autoimmune diseases may have an increased risk of developing cancer.

Autoimmune disease	Associated cancer	Risk metric (95%CI where available)	Reference
Rheumatoid arthritis	Multiple	SIR: 1.09 (1.06–1.13)	Simon et al. (2015)
Psoriasis	Multiple	RR: 1.21 (1.11–1.33)	Geller et al. (2018)
			Vaengebjerg et al. (2020)
Systemic lupus erythematosus	Multiple	RR: 1.28 (1.16–1.42)	Cao et al. (2015)
			Song et al. (2018)
Inflammatory bowel disease	Colorectal cancer	SIR: 1.7 (1.2–2.2)	Lutgens et al. (2013)
Sjogren’s syndrome	Multiple	RR: 1.53 (1.17–1.88)	Liang et al. (2014)
Autoimmune gastritis	Gastric adenocarcinoma	OR: 2.18 (1.94–2.45)	Massironi et al. (2019)
Dermatomyositis	Multiple	OR: 14.5 (2.35–89.3)	Lau et al. (2021)

### PD-1/PD-L1 Inhibitors and IrAE

There are currently six FDA-approved PD-1/PD-L1 inhibitors: nivolumab, pembrolizumab, cemiplimab, atezolizumab, durvalumab, and avelumab Table 2.

**TABLE 2:** FDA-approved PD-1/PD-L1 inhibitors (Constantinidou et al., 2019).

Target	Molecular	Antibody type	Approved in	Company	Commercial name
PD-1	Nivolumab	Human IgG4	Unresectable or metastasized melanoma; squamous non-small cell lung cancer (NSCLC); advanced renal cell carcinoma (RCC); urothelial carcinoma; colorectal cancer; hepatocellular carcinoma (HCC); head and neck cancer (HNSCC)	Bristol-Myers Squibb	Opdivo
PD-1	Pembrolizumab	Humanized IgG4	Advanced or unresectable malignant melanoma; NSCLC; HNSCC; advanced gastric cancer; Hodgkin’s lymphoma; urothelial carcinoma; bladder cancer; colorectal cancer; HCC; RCC	Merck	Keytruda
PD-1	Cemiplimab	Human IgG4	Cutaneous squamous cell carcinoma (CSCC); basal cell carcinoma; NSCLC	Sanofi, Regeneron	Libtayo
PD-L1	Atezolizumab	Humanized IgG1k	Urothelial carcinoma; NSCLC; small cell lung cancer (SCLC); breast cancer; HCC; unresectable or metastasized melanoma	Roche, Genentech	Tecentriq
PD-L1	Durvalumab	Human IgG1k	NSCLC; extensive stage-small cell lung cancer (ES-SCLC); urothelial carcinoma; bladder cancer	AstraZeneca	Imfinzi
PD-L1	Avelumab	Human IgG1	Merkel cell carcinoma (MCC); urothelial carcinoma; RCC	Merck Serono, Pfizer	Bavencio

PD-1/PD-L1 inhibitors have changed the therapeutic prospects for patients with advanced malignancies. In carcinogen-induced cancers or virus-driven cancers such as Hodgkin’s lymphoma, virus-driven skin Merkel cell carcinoma, and microsatellite instability cancer, the response rate is 50–90% (Ribas and Wolchok, 2018). The second high response rate is cancers with high immunogenicity such as melanoma, NSCLC, RCC, and HCC, and the objective response rate is between 20% and 40% (Wu et al., 2019). However, while bringing hope to patients, we also need to be alert to the unique toxicity caused by immune overactivation, that is, the emergence of immune-related adverse events (irAEs).

IrAE is very common, which can be occurred in 70% of patients treated with PD-1/PD-L1 inhibitors, and most of irAE occurs 3–6 months after the start of treatment (Michot et al., 2016). Recently, a meta-analysis (Nishijima et al., 2017) showed that PD1/PD-L1 inhibitors are associated with a lower risk of treatment-related symptoms (fatigue, anorexia, nausea, diarrhea, constipation, and sensory neuropathy) and hematological toxicity. However, in patients treated with PD1/PD-L1 inhibitors, the risk of irAE is increased. The most commonly reported irAEs are endocrine diseases (thyroid diseases such as hypothyroidism and hyperthyroidism, followed by pituitary and adrenal dysfunction), gastrointestinal tract symptoms (diarrhea, colitis, and nausea), lung disease (pneumonia), skin symptoms (rash, itch, and leukoplakia), and musculoskeletal symptoms (arthralgia and myalgia) (Khoja et al., 2017). Compared with CTLA-4 inhibitors, PD-1/PD-L1 inhibitors are more prone to pneumonia (OR 6.4; 95% CI, 3.2–12.7), hypothyroidism (OR, 4.3; 95% CI, 2.9–6.3), arthralgia (OR, 3.5; 95% CI, 2.6–4.8) and vitiligo (OR, 3.5; 95% CI, 2.2–5.3) (Igoe et al., 2020). Although irAEs are usually mild and can be controlled by clinicians, some can be fatal, such as pneumonia, cardiopulmonary arrest, heart failure, myocardial infarction, and stroke. There is evidence (Okwundu et al., 2021) that the incidence of fatal adverse events caused by immunosuppressive therapy is estimated to be 0.3%–1.3%, which is lower than the risk associated with traditional treatment, the platinum-containing dual-drug chemotherapy is about 0.9%, while the allogeneic hematopoietic stem cell transplantation is about 15%. The severity of irAE does not seem to be related to the dose of PD-1/PD-L1 inhibitors because the incidence of irAEs is very similar between 3 and 10 mg/kg nivolumab (Topalian et al., 2014). In a cohort study (Robert et al., 2014), there was no significant difference in the incidence of irAE between 2 and 10 mg/kg pembrolizumab.

Diagnosing irAE is challenging due to their high variability and nonspecific clinical manifestations (Hommes et al., 2020). This makes it complicated to distinguish irAE from other diagnoses, such as infection or tumor progression, and often leads to a delay in diagnosis, so we need to identify the specific mechanisms of irAE and biomarkers that can predict or signal irAE at an early stage. The specific mechanism of irAE is still under study. At present, its mechanism is considered to be mainly mediated by T cells, but other immune cell types have also been proposed (Sanchez et al., 2019) Figure 3.

**FIGURE 3:**
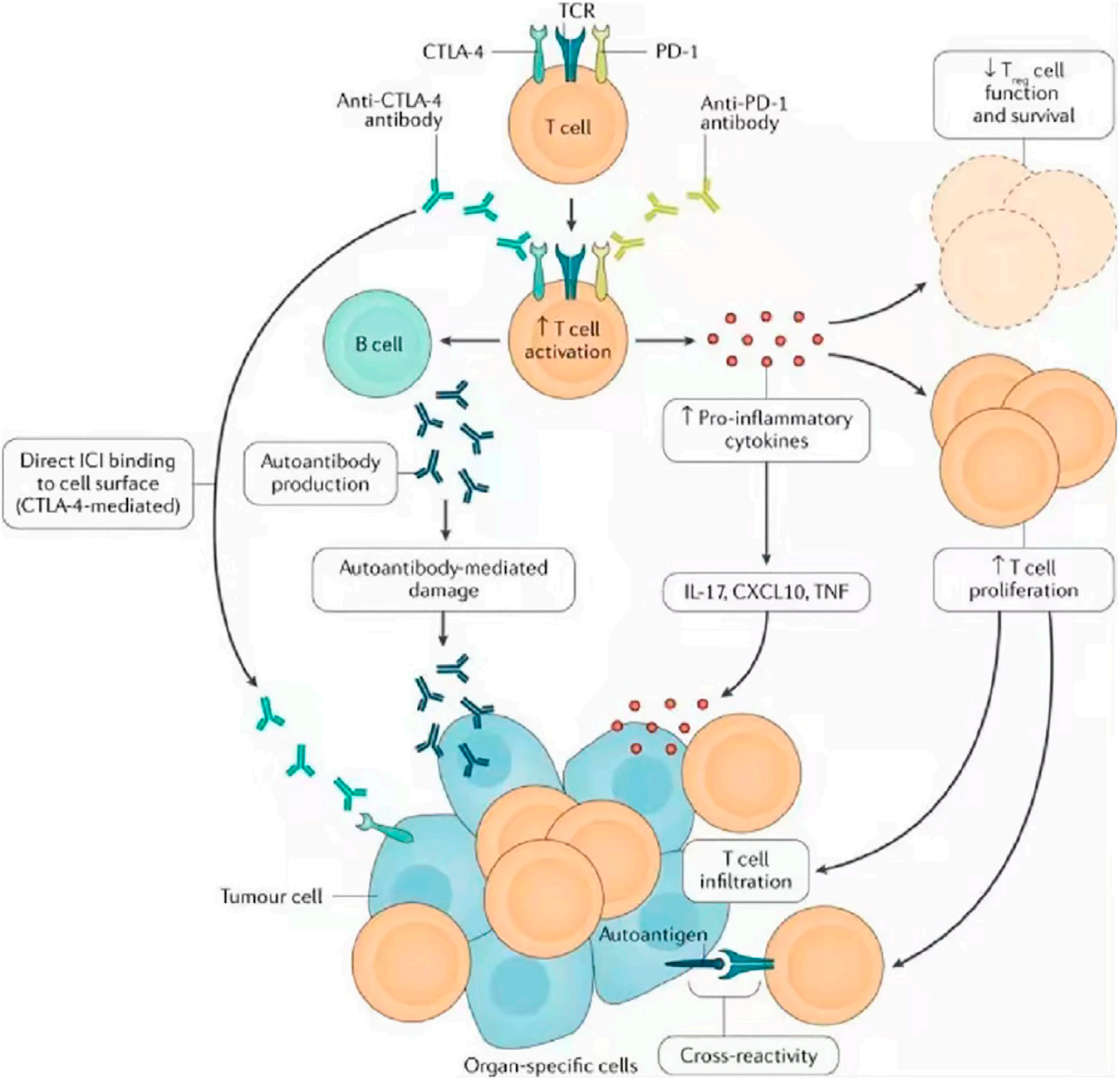
Possible mechanisms of irAE, including cross-reactions between antitumor T cells and similar antigens on healthy cells, downregulation of the function of regulatory T cells, emergence of autoantibodies, and increase of pro-inflammatory cytokines. Research on the mechanism of irAE is not only helpful in establishing treatment strategies for irAE, managing irAE and improving the prognosis and quality of life of cancer patients but also important in understanding the underlying immunology of spontaneous autoimmune diseases. TCR, T cell receptor; TNF, tumor necrosis factor. Reprinted from Ramos-Casals et al. (2020). Copyright ^©^ 2020, Springer Nature Limited.

### Cytotoxic T Cells Attack Normal Tissues

PD-1/PD-L1 inhibitors may cause the activation of cytotoxic T cells against antigens shared by tumors and normal tissues. It has been suggested that irAE may be related to epitope spreading (Lee et al., 2021). Because immunotherapy is not tumor-specific, both tumor cells and normal “bystander cells” are damaged, and neoantigens and autoantigens on tumor cells are released into the blood. Antigen-presenting cells (APCs) recognize and cross-present these antigens, triggering a secondary immune response (June et al., 2017). Epitope spreading allows T cells not only to attack more targets on the tumors but also to attack normal tissues, leading to irAE.

### Loss of Homeostasis of Regulatory T Cells

Tregs (mainly CD25^+^Foxp3^+^ Tregs) can promote immune escape of tumor cells by inhibiting antitumor immunity. PD-L1 can induce Foxp3 expression and Tregs differentiation in the periphery (Kumar et al., 2020). A current study (Wang et al., 2009) has shown that decreased intracellular Foxp3 expression was observed in the peripheral blood mononuclear cell (PBMC) of patients treated with PD-1 inhibitors for melanoma, resulting in weakened Tregs function and loss of self-tolerance, thus leading to irAE.

### B Cells and Antibody-Mediated Toxicity

A study (Das et al., 2018) has shown that immunotherapy can lead to a decrease in circulating B cells and an increase in CD21^lo^ PD1^+^ memory B cells and plasma cells. Single-cell RNA sequencing of CD21^lo^ PD1^+^ B cells showed that gene transcription related to cell activation and inflammatory cytokines increased after treatment. In addition, CD21^lo^ B cells expressed lower levels of lymphoid tissue-homing chemokine receptors CXCR4 and CXCR5 compared with CD21^hi^ B cells, suggesting that CD21^lo^ B cells may have a greater ability to transport to nonlymphoid tissues and contribute to the inflammatory process that may mediate autoimmunity (Liudahl and Coussens, 2018). Clinically, patients with early B cell changes experienced a higher incidence of irAE 6 months after treatment (Das et al., 2018). In another study (Osorio et al., 2017), when pembrolizumab was used to treat NSCLC, 21% of patients had thyroid dysfunction. Among these patients, 80% of patients developed antithyroid antibodies, and the appearance of antibodies was related to the use of PD-1/PD-L1 inhibitors. This indicates that PD-1/PD-L1 may regulate its own immune balance, leading to the rapid activation of memory B cells, recruiting classical complement and pro-inflammatory cells to signal damage. When studying the underlying mechanisms of irAE, B cell is also one of the roles that cannot be ignored.

### Cytokines

The cascade of immune responses requires effective cell-to-cell communication. Cytokines have multiple regulatory effects to maintain immune balance. However, a strong cytokine storm after immunotherapy may lead to overactivation of T cells that target self-organizations. In a prospective study (Kurimoto et al., 2020), the higher levels of serum IL-1β, IL-2, and GM-CSF as well as IL-8, G-CSF, and MCP-1 that were reduced in the early treatment period, were significantly associated with the occurrence of thyroid irAE (*p* < 0.05). In another longitudinal analysis (Lim et al., 2019), the increased expression of 11 cytokines (G-CSF, GM-CSF, fractalkine, FGF-2, IFNα2, IL12p70, IL1a, IL1B, IL1RA, IL2, and IL13) is closely related to severe irAEs which require high-dose immunomodulator intervention. Overall, in these studies, low baseline levels and after treatment significantly elevated levels of key cytokines prove that cytokines appear to be associated with irAE.

### Innate Immune Cells

In a retrospective analysis (Pavan et al., 2019), the low neutrophil-to-lymphocyte ratio (NLR) and low platelet-to-lymphocyte ratio (PLR) at baseline were significantly correlated with the occurrence of irAEs (OR, 2.2, *p* = 0.018; OR, 2.8, *p* = 0.003). In another retrospective analysis of 146 patients (Krishnan et al., 2020), patients with eosinophilia were more likely to have irAEs (*p* = 0.042). In addition, NK cells themselves may express PD-1, and the PD-1/PD-L1 axis inhibits NK cell responses *in vivo*. Therefore, treatment with PD-1/PD-L1 inhibitors may lead to the activation of NK cells, leading to potential irAEs (Hsu et al., 2018). At present, everyone is gradually beginning to focus on the specific mechanisms by which innate immune cells cause irAEs.

### Environmental Factors

More and more evidences show that individual intestinal flora can change immune homeostasis and tolerance (Young et al., 2018). The impact of antibiotic use was explored in a group of patients receiving PD-1/PD-L1 inhibitors. Compared with the nonantibiotic group, patients who received antibiotic treatment before, during, or shortly after PD-1/PD-L1 inhibitors treatment had significantly lower progression-free survival (PFS) and overall survival (OS) (Matson et al., 2018). Possible mechanisms linking microbes and immunotherapy include the stimulation of T cells by specific bacterial antigens, which then trigger cross-reactions against tumor neoantigens. Meanwhile, bacterial toxins can also stimulate the recruitment of T cells, thereby releasing inflammatory cytokines such as IL-17, and combat immune tolerance (Chau and Zhang, 2021). IrAEs may be related to bacterial metabolites, which have pro-inflammatory properties. In the case of ecological disorders, it may cause abnormal activation of the immune system Table 3. For example, short-chain fatty acids (SCFAs), the main end metabolite produced by the gut microbiota (Schirmer et al., 2016), can increase the level of IL-17, which is a pro-inflammatory cytokine that plays a key role in irAEs. It is also important to clarify the impact of non-GI tract microbiota and whether PD-1/PD-L1 inhibitors-driven irAEs is affected by other environmental factors. We still have a long way to go. It is worth mentioning that unlike PD-1 inhibitors, PD-L1 inhibitors only block the interaction between PD-1 and PD-L1 and retain the interaction between PD-1 and PD-L2. Although the overall toxicity characteristics of PD-1 inhibitors and PD-L1 inhibitors are similar, it has been suggested that targeted PD-L1 therapy reduces the frequency of irAEs to a certain extent and maintains self-tolerability (Buchbinder and Desai, 2016).

**TABLE 3:** Frequencies associated with PD-1/PD-L1 inhibitors.

Study	PD-1/PD-L1 inhibitor	Disease	Dose (n)	Any-grade adverse event (grade ≥3 adverse events)								Ref
				Diarrhea	Colitis	Pneumonia	Hepatitis	Nephritis	Endocrine	Rash	Nervous system	
Checkmate 067	Nivolumab	Stage III + Stage IV melanoma	3 mg/kg every 2 weeks (313)	22% (3%)	3% (1%)	2% (0.3%)	8% (3%)	2% (1%)	17% (2%)	24% (0.3%)	—	Hodi et al. (2018)
Checkmate 017 + 057	Nivolumab	NSCLC	3 mg/kg every 2 weeks (418)	8.9% (1%)	—	5% (1%)	6% (1%)	3% (0.2%)	9% (0)	8.1% (0.5%)	0.4% (0)	Horn et al. (2017)
												Borghaei et al. (2021)
Keynote 024	Pembrolizumab	NSCLC	200 mg every 3 weeks (154)	16.2% (3.9%)	3.9% (1.9%)	7.8% (2.6%)	0.6% (0.6%)	0.6% (0.6%)	13% (0.6%)	10.4% (1.3%)	—	Reck et al. (2019)
Keynote 158	Pembrolizumab	Non-colorectal MSI-H/dMMR cancer	200 mg every 3 weeks (233)	12% (0)	3.9% (0.9%)	3.9% (1.3%)	1.7% (0.9%)	0.9% (0)	15% (0.8%)	5.2% (0)	—	Marabelle et al. (2020)
Empower-Lung 1	Cemiplimab	NSCLC	350 mg every 3 weeks	4.2% (0.3%)	0.3% (0.3%)	0.3% (0.3%)	0.6% (0.6%)	0.3% (0.3%)	—	5% (1%)	1% (0.3%)	Sezer et al. (2021)
Oak	Atezolizumab	NSCLC	1,200 mg every 3 weeks (609)	15.4% (0.7%)	0.3% (0)	1% (0.7%)	0.3% (0.3%)	—	—	—	—	Rittmeyer et al. (2017)
Mystic	Durvalumab	NSCLC	20 mg/kg every 4 weeks (369)	1.9% (0.3%)	0.5% (0.3%)	2.2% (1.1%)	0.3% (0.3%)	0 (0)	0.8% (0.3%)	1.4% (1.1%)	—	Rizvi et al. (2020)
Javelin Merkel 200	Avelumab	Metastatic Merkel cell carcinoma	10 mg/kg every 2 weeks (88)	2.3% (0)	1.1% (0)	—	2.2% (1.1%)	1.1% (0)	6.8% (1.1%)	5.7% (0)	—	D'Angelo et al. (2020)

### Safety and Effectiveness of PD-1/PD-L1 Inhibitors in Patients With Preexisting Autoimmune Diseases

PD-1/PD-L1 inhibitors are easy to cause irAEs, and the impaired function of PD-1/PD-L1 plays an important role in a variety of autoimmune diseases (Zamani et al., 2016). Many patients with autoimmune diseases are excluded from clinical trials of PD-1/PD-L1 inhibitors because of concerns about the activation of underlying autoimmune diseases, the flare of preexisting autoimmune diseases, and the potential susceptibility to severe irAEs. However, as PD-1/PD-L1 inhibitors are used in a wider range of cancers, the need to evaluate the risk-benefit ratio of immunosuppressants used in cancer patients with preexisting autoimmune diseases will gradually increase. Recent studies (Abdel-Wahab et al., 2018; Danlos et al., 2018; Cortellini et al., 2019; Tison et al., 2019; Xie et al., 2020) have shown that the risk of the flare of autoimmune diseases or *de novo* irAEs after receiving immunotherapy is statistically higher in patients with preexisting autoimmune diseases, but they are usually mild and can be controlled without stopping the drugs. With close monitoring of patients’ symptoms and multidisciplinary cooperation, PD-1/PD-L1 inhibitors are relatively safe (Menzies et al., 2017; Pantuck et al., 2019). Next, we will summarize the data from several of these studies Table 4.

**TABLE 4:** Retrospective studies of PD-1/PD-L1 inhibitors in patients with preexisting AID.

Author	Patient	Tumor	AID exacerbation	*De novo* irAE	ORR
Menzies	N = 52, rheumatoid arthritis (13), other rheumatic diseases (14), psoriasis (6)	Melanoma	20 (38%)	15 (29%)	17 (33%)
Menzies et al. (2017)					
Alice Tison	N = 112 (PD-1/PD-L1 inhibitors n = 95), psoriasis (31), rheumatoid arthritis (20), and inflammatory bowel disease (14)	Melanoma (66), NSCLC (40), urinary cancer (4), MCC (2)	43 (45%)	34 (36%)	48 (52%)
Tison et al. (2019)					
Leonardi	N = 56, rheumatism (25), psoriasis (14), and endocrine diseases (9)	NSCLC	13 (23%)	21 (38%)	11 (22%)
Leonardi et al. (2018)					
Hoa	N = 27, rheumatoid arthritis (8), psoriatic arthritis (8), and inflammatory bowel disease (4)	NSCLC (15), melanoma (8), other cancers (4)	14 (52%)	14 (52%)	14 (52%)
Hoa et al. (2021)					
Monique	N = 187, rheumatism (89), endocrine diseases (73), and IBD (31)	Melanoma	—	Grade 3 or 4 irAE, 31 (17%)	71 (40%)
van der Kooij et al. (2021)					
Gutzmer	N = 19, rheumatism (9), thyroiditis (5), and psoriasis (2)	Melanoma	8 (42%)	3 (16%)	6 (32%)
Gutzmer et al. (2017)					
Loriot	N = 35, psoriasis (15), thyroid disease (6), and rheumatoid arthritis (4)	Urinary system cancers	4 (11%)	—	4 (11%)
Loriot et al. (2020)					
Fountzilas	N = 123 (PD-1 inhibitors *n* = 102), rheumatism (54) and endocrine diseases (26)	NSCLC (84), melanoma (18), head and neck cancer (6)	31 (25.2%)	43 (35%)	57 (56.4%)
Fountzilas et al. (2022)					
Richter	N = 16 (PD-1 inhibitors *n* = 12), rheumatism (16)	Melanoma (10), pulmonary (4), hematologic (2)	1 (6.3%)	6 (38.5%)	—
Richter et al. (2018)					

In a systematic review (Abdel-Wahab et al., 2018) of 123 cancer patients with preexisting autoimmune diseases, after treatment with PD-1/PD-L1 inhibitors, 92 (75%) patients had exacerbations of preexisting autoimmune diseases (41%), *de novo* irAEs (25%), or both (9%). In patients with active and inactive autoimmune diseases, no difference in adverse events was observed. It is interesting to note that CTLA-4 inhibitors are more likely to cause *de novo* irAEs, while PD-1/PD-L1 inhibitors are relatively more likely to cause the exacerbation of autoimmune diseases. In another article (Xie et al., 2020) that covers 619 AID patients receiving immunotherapy, 60% of patients had different degrees of exacerbation of the original autoimmune disease (27%), *de novo* irAEs (25%), or both (8%). Most *de novo* irAEs are mild, occurring most commonly in colitis, thyroiditis, and hypophysitis. Interestingly, compared with other autoimmune diseases, patients with rheumatoid arthritis seem to tend to aggravate the onset of the disease (RR = 1.25–1.88). This suggests that the type of AID may have heterogeneity in the safety of patients.

In a prospective study (Danlos et al., 2018), 45 cancer patients with 53 AIDs were evaluated and compared with 352 non-AID patients included in the same period. The study found that 20 patients (44.4%) had at least one irAE, while 102 (29%) of non-AID patients had irAE. More than half of AID patients did not have a disease attack, and only 25% of irAE patients were in the need to stop PD-1 inhibitors. There was no significant difference between the AID group and the non-AID group in terms of overall survival time and objective response rate (*p* = 0.38 and 0.098), indicating that PD-1/PD-L1 inhibitors seem to be safe and effective in patients with AID as in patients without AID.

In a “real world” retrospective multicenter observational study (Cortellini et al., 2019), 56 (65.9%) and 8 (9.4%) patients experienced any grade of irAEs and grade 3/4 of irAEs, respectively. In contrast, among 666 non-AID patients, 266 (39.9%) and 59 (8.8%) patients had experienced any grade of irAEs and grade 3/4 of irAEs, respectively. This indicates that patients with preexisting AID have a significantly higher risk of irAEs (*p* < 0.0001), but they do not seem to be exposed to the risk of serious adverse events (*p* = 0.8863).

In summary, although the risk of irAEs is increased, most irAEs are mild and controllable. For most patients with AID, clinicians should consider the potential severity of AID in patients before administering treatment, properly inform patients of the risks and benefits of treatment, and these patients should be closely monitored during and after treatment. Under multidisciplinary cooperation and close monitoring, the use of PD-1/PD-L1 inhibitors may be not only safe but also effective (Rakshit and Molina, 2020). However, most of the evidences for the use of PD-1/PD-L1 inhibitors in preexisting AID patients are limited to retrospective analysis with associated risk of bias, such as selection bias. They also had a relatively small sample size, which could limit the validity of the results. Larger retrospective and prospective analyses will help further to characterize the risk of immunotherapy for patients with specific autoimmune diseases.

### AID Types and Patients Safety

Tison et al. (2019) found that autoimmune diseases flare in patients receiving PD-1/PD-L1 inhibitors after treatment varies depending on the type of preexisting autoimmune disease. It is more common in patients with psoriatic/psoriatic arthritis or RA than in patients with lupus. The onset of inflammatory bowel disease is the most serious, and three patients require biological DMARD, so we must be careful with these patients. Fountzilas et al. (2022) found that patients with flare had more commonly underlying dermatologic diseases (*n* = 12, 38, 7%), with the vast majority (*n* = 10) having been diagnosed with psoriasis. Gutzmer et al. (2017) pointed out that the flare of AID seems to be more common in patients with rheumatism and psoriasis. Menzies et al. (2017) found that 14/27 (52%) patients with rheumatism, 3/8 (37.5%) patients with psoriasis, 1/4 (25%) patients with Graves’ disease, and 2/2 (100%) patients with immune thrombocytopenic purpura developed autoimmune diseases flare, and in contrast, patients with gastrointestinal (*n* = 6), nervous system (*n* = 5), and respiratory (*n* = 2) diseases did not experience flare after treatment. Leonardi et al. (2018) reported that the flare of rheumatism was significantly higher than that of non-rheumatism AID patients (40% vs. 10%; *p* = 0.01). Xie et al. (2020) found that compared with Pso/PsA (RR, 1.25; 95% CI, 0.85–1.82), AIT (RR, 1.88; 95% CI, 0.92–3.85), or IBD (RR, 1.50; 95% CI, 0.86–2.63), the risk of flare of RA is numerically higher, but it does not reach statistical significance. At the same time, the study puts forward the trend of more flares in patients with rheumatism. In terms of mechanism, studies (Zamani et al., 2016) have shown that the single nucleotide polymorphism of the PD-1 encoding gene *PDCD1* may be related to the flare of autoimmune diseases. The polymorphism of intron 4 (PD1.3) interferes with the binding of runt-related transcription factor 1 (RUNX1) and affects the production of PD-1. There is a statistically significant association between the SNP at the RUNX1 binding site and the susceptibility to RA and psoriasis (Lee et al., 2009), which easily leads to the appearance of RA and psoriasis, while many other AIDs do not involve or rely heavily on the PD-1 signal path. This suggests that doctors should also consider AID types (Pantuck et al., 2019) when deciding whether to use PD-1/PD-L1 inhibitors. Compared with other autoimmune diseases, for patients with rheumatism or psoriasis, we should be more alert to the flare of original autoimmune diseases after treatment and should carefully weigh the benefits and risks of treatment. Due to the complexity and diversity of possible AID flares and irAEs, multiple sub-specialist physicians need to cooperate to care for these patients.

### AID Status and Patients Safety

The intensity of the autoimmune response is often closely related to the condition. When the condition is active, the autoantibody titers increase, and when the condition is inactive, the autoantibody titers decrease. Therefore, we speculate that the state of autoimmune diseases can affect the immune system, and there seems to be a correlation with the incidence of adverse events in the immunotherapy of cancer patients. In a retrospective study of 751 stage IV cancer patients by Cortellini et al. (2019), in inactive AID, the incidence of any grade of irAEs and G3/G4 irAEs was 64.3% and 8.6%, respectively. In active AID, the incidence of any grade of irAEs and G3/G4 irAEs was 73.3% and 13.3%, respectively. The ORR of patients with preexisting inactive and active AID was 38.1% (95% CI, 24.4–56.6) and 50% (95% CI, 23.0–76.9), and the median PFS was 14.4 months (95% CI, 5.3–17.1) and 6.8 months (95% CI, 5.1–9.4), and the median OS was 15.7 months (95% CI, 10.3–24.3) and 9.8 months (95% CI, 5.8–24.6), respectively. Compared with inactive AID, patients with active AID have relatively higher irAEs and higher ORR but lower PFS and OS, which may be related to the increased morbidity and mortality of AID itself. Menzies et al. (2017) reported that symptomatic patients (9/15, 60%) had more frequent disease flares and exacerbations than clinically inactive AID patients (11/37, 30%) (*p* = 0.039). Leonardi et al. (2018) retrospectively analyzed 56 AID patients with advanced NSCLC and found that the exacerbations of the original AID in symptomatic patients (*n* = 5, 50%) were significantly higher than those in asymptomatic AID patients (*n* = 8, 18%; *p* = 0.04). This indicates that the risk of irAEs for active AID is relatively higher. For safety reasons, it may be necessary to correctly treat and control severe active AID before immunotherapy starts. We are still unable to determine the best strategy for providing PD-1/PD-L1 inhibitors in patients with active or symptomatic AD, which presents a huge unmet medical need for the medical oncology community. Currently, there are no consensus guidelines for treatment for this particular patient population, nor is there a way to determine the risk-benefit ratio for individual patients. We strongly recommend that patients be aware of possible complications and corresponding comorbidities associated with treatment. Treatment can only be carried out after patient education and doctor–patient communication. Static AID patients can be regarded as non-AID patients (Danlos et al., 2018), but we still need a multidisciplinary team to closely monitor these patients to identify some predictable adverse events and provide consultation to patients.

### Relationship Between IrAE and Treatment Efficacy

More and more literatures (Grangeon et al., 2019; Hussaini et al., 2021) indicate that there is a potential correlation between the onset of irAE and the efficacy of PD-1/PD-L1 inhibitors. Among patients with NSCLC, Hussaini et al. (2021) summarized 19 retrospective studies and found that ORR (irAE^+^ irAE^−^) was 41.49% (95% CI, 36.5–46.5) and 18.01% (95% CI, 13.5–22.6), the weighted average PFS was 8.97 months (95% CI, 7.14–10.8) and 3.06 months (95% CI, 2.4–3.72), OS (irAE^+^ irAE^−^) was 19.07 months (95% CI, 14.3–23.8) and 7.45 months (95% CI, 5.34–9.56), respectively. In another observational study (Grangeon et al., 2019) of 270 patients who had received PD-1/PD-L1 inhibitors, 44% of patients experienced any grade of irAEs. Compared with patients who did not experience irAEs, patients who experienced irAEs had a higher PFS (5.2 vs. 1.97 months, *p* < 0.001), ORR (22.9% vs. 5.7%, *p* < 0.0001), and disease control rate (DCR) (76% vs. 58%, *p* < 0.001). In patients with metastatic melanoma, multiple studies (Sanlorenzo et al., 2015; Hua et al., 2016) have shown that leukoplakia, a clinically visible irAE, may be related to the clinical benefit of PD-1/PD-L1 inhibitors. For example, in a prospective study (Hua et al., 2016) of melanoma patients receiving pembrolizumab, the remission rate of patients with vitiligo was 71%, while the remission rate of patients without vitiligo was 28%. In another retrospective analysis (Weber et al., 2017) of 576 patients with advanced melanoma treated with nivolumab monotherapy, the ORR of patients who experienced irAEs of any grade was significantly better than that of patients who did not experience them (48.6% vs. 17.8%, *p* < 0.001). A retrospective analysis (Verzoni et al., 2019) of 389 patients receiving nivolumab for advanced or metastatic RCC showed that patients with irAEs had a more significant survival benefit than patients without irAEs (median OS, not reach and 16.8 months, *p* = 0.002; 1-year OS, 75.4% and 59.8%; 2-year OS, 66.9% and 36.8%).

In summary, it is not difficult to find that in patients receiving PD-1/PD-L1 inhibitors, the occurrence of irAEs is positively correlated with the efficacy. Of course, one of the possible deviations is the duration of treatment. Its increase will lead to prolonged drug exposure, which leads to a higher probability of adverse events and better treatment results. In order to minimize the deviation of drug treatment time, Verzoni et al. (2019) conducted a landmark analysis of OS at the median time (6 weeks) of the appearance of irAEs and found that irAEs were still statistically significantly associated with improvement in OS (*p* = 0.006). In another study (Schadendorf et al., 2017), the authors evaluated the efficacy during the induction phase between patients who discontinued due to irAEs (*n* = 96) and patients who did not discontinue with irAEs (*n* = 233) (median duration of treatment was 1.4 and 9.4 months, respectively). The ORR of patients who discontinued the drug due to irAEs was 58.3%, the median PFS was 8.4 months, and the OS rate at 18 months was 67%, while the ORR of patients who did not discontinue the drug was 50.2%, and the median PFS was 10.8 months, and the OS rate at 18 months was 62%. The PFS, OS, and ORR between the two groups seem to be similar, indicating that the duration of the drug treatment is not the cause of the relationship between irAEs and drug efficacy.

Interestingly, different literatures have different results regarding the relationship between the severity of irAEs and the efficacy. IrAE is thought to be mainly mediated by T cells. Antigen-sharing or cross-reactivity leads to T cell-mediated responses not only against tumor cells but also against healthy cells. In terms of mechanism, it seems that the more severe the irAEs, the higher the activity of T cells, and therefore the better the efficacy of the drug. In a retrospective study (Fujii et al., 2018), 98 of 290 patients (34%) experienced any grade of irAEs, and 15 (5.2%) experienced grade 3 or higher irAEs. Compared with patients with irAEs below grade 3, patients with grade 3 irAEs had high ORR (25% vs. 6%; *p* = 0.039) and long median progression time (30 vs. 10 weeks; *p* = 0.0040). However, through multivariate analysis, Judd et al. (2017) found that low-grade irAEs have a higher ORR (*p* = 0.017), which may be related to the lower incidence of high-grade irAEs and the inability to detect its association through a small sample. In a retrospective analysis of 576 patients with advanced melanoma, Weber et al. (2017) found that patients with ≥grade 3 irAEs had no significant difference in ORR compared with other patients. The different conclusions may be related to the high-grade irAEs may lead to death, which confuses the difference in survival. In addition, severe toxicity is usually associated with more aggressive immunosuppression, which may also affect the efficacy (Das and Johnson, 2019). Future studies with larger sample sizes are needed to investigate the relationship between irAE severity and efficacy.

### Efficacy and Safety of Baseline Immunosuppressive Therapy

Since the age of immunotherapy, corticosteroid therapy has been considered an antidote to irAE (Rossi et al., 2019). For patients with active autoimmune diseases, corticosteroid drugs also seem to be necessary. Mechanistically, the use of PD-1/PD-L1 inhibitors, combined with baseline immunosuppressive drugs, appears to improve AID symptoms and irAEs, but on the other hand, whether it will reduce the efficacy of patients is still discussed. Menzies et al. (2017) found that the response rate of immunosuppressive drugs (3/20, 15%) at the beginning of treatment was lower than that of unused immunosuppressive drugs (14/32, 44%) (*p* = 0.033). After adjusting for prognostic factors (AJCC stage, brain metastases, ECOG PS, and LDH), it was still significant (*p* = 0.029). However, there were more AID flares after baseline immunosuppressive therapy at using PD-1 inhibitors (10/20, 50%) than without immunosuppressive therapy (10/32, 31%). Xie et al. (2020) indicated that the ORR of patients receiving immunosuppressive therapy is lower than that of patients not receiving immunosuppressive therapy, but this is not statistically significant (RR, 0.58; 95% CI, 0.26–1.33). In a national multicenter cohort study (Tison et al., 2019), Tison found that patients who received immunosuppressive therapy at the beginning of immunotherapy had a shorter median PFS (3.8 vs. 12 months; *p* = 0.006), but there was no significant difference in OS. Similarly, Fountzilas et al. (2022) observed an association between corticosteroid-treated AID patients at initiation of immune checkpoint inhibitors and shorter PFS (HR = 2.08, 95% CI, 1.18–3.68, Wald’s *p* = 0.012). However, he found that the initiation of immunotherapy with immunomodulators (excluding corticosteroids) was not associated with PFS (*p* = 0.22). However, in the smaller case series reported by Leonardi et al. (2018), Gutzmer et al. (2017), no negative effects on tumor response were found. In a case review (Lipson et al., 2016), PD-1 inhibitors were administered to solid organ transplant recipients with metastatic squamous cell carcinoma of the skin (standard long-term immunosuppressive therapy). The patient has a strong antitumor response and rejection of allogeneic transplantation, indicating that PD-1 inhibitors can be very effective against cancer in the context of chronic immunosuppression. A recently published retrospective study by Ricciuti et al. (2019) showed that the worse ORR, PFS, and OS observed in patients receiving ≥10 mg prednisone appeared to be related to the indications for corticosteroid use. When corticosteroids are used in cancer-related palliative care, the prognosis is worse. However, when corticosteroids are used in the treatment of indications unrelated to cancer, such as autoimmune diseases, compared with patients receiving 0–10 mg prednisone, there were no significant differences in mPFS or mOS among patients receiving ≥10 mg prednisone for cancer-unrelated indications. In summary, the existing research may mainly support that it is not necessary to stop immunosuppressants such as corticosteroids before initiation of immunotherapy in AID patients with cancer because immunosuppressants can inhibit AID and irAEs and may have little effect on immune efficacy. In view of the small sample size of existing studies and the heterogeneity between patients, more prospective studies are needed in the future to clarify conflicting data regarding the use of PD-1/PD-L1 inhibitors in patients with preexisting AID. We hoped to have more data to study the efficacy of PD-1/PD-L1 inhibitors in patients with active AID not on therapy, AID controlled on therapy, and AID off therapy at time of initiation of immunotherapy (Dietz et al., 2021). We raised the issue that corticosteroids differ in efficacy from other immunomodulators, and that immunotherapy in patients treated with corticosteroids needs to be treated with caution.

### Efficacy and Safety of Gender

Men and women have different immune responses to foreign and self-antigens and show differences in innate and adaptive immune responses (Klein and Flanagan, 2016). These gender-based immunological differences lead to changes in the incidence of autoimmune diseases, the response rate of immunotherapy, and relevant adverse events. Most autoimmune diseases are more common in women (Ngo et al., 2014). However, whether gender affects the efficacy and safety of immunotherapy remains controversial. Cortellini et al. (2019) found that in cancer patients with AID, women had a significant increase in irAEs after immunotherapy. Compared with men, the risk of irAEs was 1.4 times high. They speculated that the higher incidence of irAEs in women may be related to longer OS. However, in a recent meta-analysis of 1096 female patients and 1886 male patients, Jing et al. (2021) found that there was no statistically significant difference in irAEs between the sexes (OR = 1.19, 95% CI, 0.91–1.54, *p* = 0.21). When (Shah et al., 2020) performed univariate or multivariate analysis of 455 melanoma patients receiving PD-1/PD-L1 inhibitors, they found that gender was not related to the incidence of any irAEs, severe irAEs, or hospitalization. In terms of efficacy, Conforti et al. (2018) reported that immune checkpoint inhibitors can significantly improve the OS of patients, but compared with women, these drugs have a great therapeutic effect on men. For example, the reduction in the risk of death is twice as large in men as in women. They believe that the reason for the difference may be that women have stronger immunity than men (Klein and Flanagan, 2016). This means that female-developed tumors must escape more effective immune surveillance mechanisms, which can make advanced female tumors less immunogenicity and stronger ability to escape immunity than similar tumors in men, so they may be more resistant to immunotherapy. In contrast, Wallis et al. (2019) conducted a systematic review of 18 randomized clinical trials of PD-1 inhibitors for advanced solid cancers, including 7,198 men and 3,495 women. They found no statistically significant differences between genders (I^2^ = 44%; *p* = 0.94). Botticelli (Botticelli et al., 2017) also reported that for OS (male and female, HR, 0.72; 95% CI, 0.64–0.83 and HR, 0.81; 95% CI, 0.70–0.94, *p* = 0.285) and PFS (male and female, HR, 0.66; 95% CI, 0.52–0.82 and HR, 0.85; 95% CI, 0.66–1.09, *p* = 0.158), although the HR of men is lower than that of women, it is not statistically significant. Ye et al. (2020) believed that the aforementioned meta-analyses are simply a collection of different clinical trials, which may not provide clear results. They found that male patients with colorectal cancer (*p* = 0.041) or glioblastoma multiforme (*p* = 0.011) showed better OS with PD-1/PD-L1 inhibitors treatment. Female patients with esophageal gastric cancer or NSCLC tend to have better OS. It was further observed that compared with male patients with NSCLC (6/24, 25%), female patients (16/32, 50%) had a high response rate, indicating that the immune characteristics of different cancer types are closely related to gender (such as male bias in melanoma and female bias in lung squamous cell carcinoma). In summary, how gender affects cancer immunotherapy is still a key gap for us because it is related to precision medicine (Zhu and Boutros, 2021). Current retrospective studies related to gender are mixed with a large number of confounding factors, such as cancer types, race, and age. Instead of focusing only on the two variables of gender and efficacy, future research should control for variables such as cancer types, race, and age. How gender affects the cancer genome and molecular data related to immunotherapy should be something we need to pay attention to in the future.

### Prevention and Management of IrAEs in AID Patients

The current consensus is that the best management of irAEs mainly depends on early identification to reduce the possibility of discontinuation of treatment, ensure the quality of life, and avoid or minimize the risk of rare fatal results (Martins et al., 2019). However, there is still a lack of biomarkers that can individually assess the risk of irAEs. In terms of internal factors, Hoefsmit et al. (2019) believed that the similarity of irAEs and autoimmune diseases supports the hypothesis that irAEs may be related to the susceptibility gene loci of various autoimmune diseases. It is also written previously that the use of PD-1/PD-L1 inhibitors in AID patients has an increased risk of irAEs, especially in patients with previous rheumatism and psoriasis as well as active AID diseases. Gender seems to also affect the onset of irAEs. In terms of external factors, the combination of PD-1/PD-L1 inhibitors and CTLA-4 inhibitors has a higher overall incidence of irAEs than monotherapy (Larkin et al., 2015). In addition, a literature (Kang et al., 2021) suggests that changes in cytokine concentration before and during immunotherapy may help early to predict the risk of irAEs in cancer patients. Nishino et al. (2016) found that the incidence of PD-1 inhibitors-related pneumonia in patients with NSCLC, RCC, and during combined treatment was higher, which was significantly higher than that of melanoma. This suggests that there seems to be a certain connection between the types of cancer and irAEs. The risk of specific irAE is increased. Meantime, clinicians began to pay attention to the relationship between the early increase of autoantibodies and irAE. Yoon et al. (2021) found that baseline anti-TPO antibody positivity and new development of anti-Tg antibody positivity during the therapy were significantly associated with the progression to hypothyroidism. In a cohort study (Toi et al., 2019) of 137 patients with advanced NSCLC treated with nivolumab or pembrolizumab, patients with classic autoreactive antibodies (such as ANA, rheumatoid factor, and antithyroid antibodies) had a higher incidence of irAEs (OR, 3.25, 95% CI, 1.59–6.65, *p* = 0.001), but they had higher PFS (6.5 months, 95% CI, 4.4–12.9 vs. 3.5 months, 95% CI, 2.4–4.1). Gowen et al. (2018) analyzed autoantibodies in patients with metastatic melanoma using high-throughput protein arrays, and their data showed that measuring patients’ serum autoantibodies could predict the development and severity of irAEs. The likely mechanism is that PD-1/PD-L1 blockade or deletion of PD-1 can lead to an augmented B cell proliferative and antibody response to T cell-independent antigens as well as enhanced IgG isotype switching and longevity (Boland et al., 2020). These studies suggest a mechanism by which PD-1 blockade could lead to autoantibody expansion and subsequent irAE development. The assessment of relevant risk factors will help to identify patients that clinicians need to be highly vigilant or even unsuitable for immunotherapy, and help clinicians determine the risk-benefit ratio of individual patients to maximize the benefits of treatment while minimizing severe toxicity. It is worth mentioning that although AID patients are at high risk for irAEs, they are not contraindications to PD-1/PD-L1 inhibitors. The use of immunotherapy with caution may be acceptable, but the recurrence of underlying autoimmune diseases should be closely monitored at the same time (Brahmer et al., 2018).

The American Society of Clinical Oncology (ASCO) (Brahmer et al., 2018), the Society for Immunotherapy of Cancer (SITC) (Puzanov et al., 2017), the National Comprehensive Cancer Network (NCCN) (Thompson et al., 2020), and the European Society for Medical Oncology (ESMO) (Haanen et al., 2017) have issued management recommendations for irAE. These recommendations may be applicable to patients with preexisting autoimmunity disease (Tison et al., 2019). The recommended treatment usually includes topical or systemic steroids as first-line treatment. In certain cases, other drugs such as infliximab, mycophenolate mofetil, or cyclophosphamide may be recommended (Ramos-Casals et al., 2020). For example, (Brahmer et al., 2018), for grade 1 irAE, ASCO recommends continuing immunotherapy and monitoring closely. For grade 2 irAE, ASCO recommends temporarily stopping treatment, using moderate systemic steroids (0.5–1 mg/kg/d prednisone), and restarting immunotherapy when the toxicity drops to grade 1 or the symptoms disappear. For grade 3 irAE, ASCO recommends using high-dose steroids (1–2 mg/kg/d prednisone), gradually reducing the dose within 4–6 weeks, and adding immunosuppressive agents in some refractory cases. For grade 4 irAE, ASCO recommends permanent discontinuation of PD-1/PD-L1 inhibitors. With proper management, most irAEs will be resolved (Friedman et al., 2016). For AID patients, it has been discussed previously that although their risk of irAEs is increased, their toxicity is usually controllable. In the study of Danlos (Danlos et al., 2018), 20 subjects with preexisting autoimmune diseases developed irAEs, and only 25% needed to stop treatment. In another study (Gutzmer et al., 2017), adverse events were successfully controlled by immunosuppression and symptom management, and none of the patients required discontinuation of PD-1/PD-L1 inhibitors. The efficacy of PD-1/PD-L1 inhibitors with baseline corticosteroids remains controversial. In AID patients, long-term use of corticosteroids is likely to cause some drug-related adverse events (Puzanov et al., 2017), such as opportunistic infections, sleep disorders, gastritis, and even diabetes and osteoporosis. For active AID, proper control and treatment are required before immunotherapy starts. In this regard, Haanen et al. (2020b) proposed a two-step strategy. First, in order to reduce the risk of impairing the efficacy before the start of immunotherapy, non-selective immunosuppressants (corticosteroids, mycophenolate mofetil, cyclophosphamide, and MTX) can be replaced by specific selective immunosuppressive drugs [RTX (anti-CD20), VDZ (anti-α4β7 integrin), and TCZ (anti-IL-6), anti-IL-12/23]. After 2–4 weeks, the combination of PD-1/PD-L1 inhibitors and selective immunosuppressants can prevent the deterioration of AID. Several documents seem to support the feasibility of this strategy. Dimitriou et al. (2021) believed that anti-IL6 therapy is effective in treating irAEs or preventing the flare of autoimmune diseases. Among 22 patients (20 patients received irAE treatment and 2 patients received the prophylactic treatment), 21 patients achieved clinical improvement and were well tolerated, and 11 (50%) patients experienced self-limiting and transient toxicity. Frohne et al. (2019) introduced a case of melanoma with IBD. The patient was treated with vedolizumab (anti-α4β7), and his IBD continued to remission. At the same time, pembrolizumab was used to successfully treat metastatic melanoma. With appropriately targeted immunotherapy, patients with preexisting autoimmune diseases can continue to receive immunosuppressive therapy and also receive immune checkpoint inhibitors therapy. Another case report (Uemura et al., 2016) also showed that PD-1/PD-L1 inhibitors combined with selective immunosuppressive drugs can bring clinical benefits. It may delay the deterioration of autoimmunity in patients with advanced melanoma and Crohn’s disease, while the antitumor effect is not affected. UC (ulcerative colitis) patients with breast cancer can also be well controlled by anti-TNF therapy without tumor progression (Ben Musa et al., 2014). According to the literature (Yasunaga, 2020), IL-7R signaling plays an important role in the development and progression of lymphoid malignancies and autoimmune diseases, and the abnormal homing activity and steroid resistance caused by IL-7R signaling may worsen the prognosis. Therefore, anti-IL-7R-targeted antibody therapy may be beneficial in the treatment of these two diseases. These indicate that in patients with preexisting active AID, it seems that selective immunosuppressive agents can also be used with caution, but this conclusion still needs more prospective studies to verify. In addition, the combination of tumor-targeted delivery with the continuous expression and release of checkpoint molecules allows these inhibitors to be targeted to desired cells, thus improving efficacy and avoiding toxicity and off-target effects. Research interest in nanomedicine is shifting rapidly toward the adaptation of delivery platforms for improving the percentage of patients who derive clinical benefit from PD-1/PD-L1 inhibitors (Martin et al., 2020). Nanomedicine can reduce, but not eliminate, the risk of certain life-threatening toxicities. In a Phase III trial (NCT02425891), atezolizumab combined with nab-paclitaxel extended progression-free survival in patients with metastatic triple-negative breast cancer (Schmid et al., 2018), suggesting that nab-paclitaxel can enhance the anticancer activity of atezolizumab. The use of lipid-based nanodrugs to deliver vaccines to promote antitumor immunity is the focus of preclinical and clinical research (for example, NCT02410733). In addition to nanomaterials, different tumor-targeted delivery vehicles are under development, which include, but are not limited to, viral vectors, platelets or hematopoietic stem cells, DNA-encoded monoclonal antibodies, bacteria, injectable hydrogels, and matrix-binding checkpoint inhibitor conjugates (Lamichhane et al., 2019). We can use nanodrug delivery platforms with specific targeting properties for each component of the tumor microenvironment. The tumor microenvironment of targeted nanomedicine can reshape the immunosuppressive tumor microenvironment into a state of immune stimulation and enhance the immune response at the tumor site and improve the anticancer effect through immune checkpoint blocking combination therapy (Kim et al., 2021). Yasunaga (2020) had a vision for the future. He believed that next-generation antibody therapies, such as antibody–drug conjugates and bispecific antibodies (bsAbs), would have a promising application prospect in cancer patients with preexisting AID.

In addition, in the context of the increased probability of irAEs in AID patients, it is inevitable that many patients will stop using immunologic drugs due to serious adverse events during PD-1/PD-L1 inhibitors treatment. When the relevant irAEs are relieved, whether to continue to use PD-1/PD-L1 inhibitors and how to balance the clinical benefits and related toxicity of each patient become more and more challenging (Haanen et al., 2020a). In a retrospective analysis (Santini et al., 2018), among 482 NSCLC patients who received PD-L1 inhibitors, 68 (14%) had serious irAEs that required interruption of treatment. Among them, 38 (56%) patients were retreated, and 30 (44%) patients stopped treatment. In the retreatment cohort, 18 (48%) patients had no follow-up irAEs, 10 (26%) patients had initial irAEs recurrence, and 10 (26%) patients had *de novo* irAEs. Most recurrences/*de novo* irAEs were mild (58% were grade 1–2) and controllable (84% resolved or reduced to grade 1). In patients with no improvement in symptoms before the occurrence of irAEs, PFS and OS in the retreatment cohort were longer. In contrast, for those patients who had an objective response before irAEs, PFS, and OS in the retreatment and discontinuation cohorts were similar. Similarly, another retrospective analysis (Schadendorf et al., 2017) showed that many patients continued to benefit from previous immunotherapy even after the drugs were discontinued due to irAEs. ASCO believes that for patients who have not responded yet or have insufficient response, it is reasonable to consider resuming treatment after the toxicity is resolved. However, if the patient has achieved an objective response when the irAEs appear, the response is likely to be long-lasting, and it is not recommended to resume treatment because it comes with a risk of recurrence of toxicity.

## Conclusion

Although there is a lack of prospective studies on the efficacy and safety of PD-1/PD-L1 inhibitors in cancer patients with preexisting AID, most retrospective analyses show that the efficacy of PD-1/PD-L1 inhibitors in AID patients is similar to that in the general population, and most of the irAEs were mild and controllable. The clinical manifestations of irAEs in AID patients are complex and diverse, which requires clinical oncologists to increase their awareness of such patients, encourage multidisciplinary cooperation and interaction, and monitor patients individually according to the types of AID, the state of AID, cancer types, treatment drugs, and even gender. In addition, we also need to pay attention to basic research work to figure out the mechanism of irAEs and its relationship with autoimmune diseases. Only by understanding these, can many problems be solved, such as the biomarkers that are helpful for identifying irAEs and targeted therapy drugs in AID patients. The ultimate goal is to maximize the benefits of antitumor response for cancer patients with preexisting AID while minimizing the risk of irAEs.
